# HGDTI: predicting drug–target interaction by using information aggregation based on heterogeneous graph neural network

**DOI:** 10.1186/s12859-022-04655-5

**Published:** 2022-04-12

**Authors:** Liyi Yu, Wangren Qiu, Weizhong Lin, Xiang Cheng, Xuan Xiao, Jiexia Dai

**Affiliations:** 1 School of Information Engineering, Jingdezhen Ceramic Institute, Jingdezhen, China; 2 School of Foreign Languages, Jingdezhen University, Jingdezhen, China

**Keywords:** Drug–target interaction, Graph neural network, Molecular fingerprint, Pseudo amino acid composition

## Abstract

**Background:**

In research on new drug discovery, the traditional wet experiment has a long period. Predicting drug–target interaction (DTI) in silico can greatly narrow the scope of search of candidate medications. Excellent algorithm model may be more effective in revealing the potential connection between drug and target in the bioinformatics network composed of drugs, proteins and other related data.

**Results:**

In this work, we have developed a heterogeneous graph neural network model, named as HGDTI, which includes a learning phase of network node embedding and a training phase of DTI classification. This method first obtains the molecular fingerprint information of drugs and the pseudo amino acid composition information of proteins, then extracts the initial features of nodes through Bi-LSTM, and uses the attention mechanism to aggregate heterogeneous neighbors. In several comparative experiments, the overall performance of HGDTI significantly outperforms other state-of-the-art DTI prediction models, and the negative sampling technology is employed to further optimize the prediction power of model. In addition, we have proved the robustness of HGDTI through heterogeneous network content reduction tests, and proved the rationality of HGDTI through other comparative experiments. These results indicate that HGDTI can utilize heterogeneous information to capture the embedding of drugs and targets, and provide assistance for drug development.

**Conclusions:**

The HGDTI based on heterogeneous graph neural network model, can utilize heterogeneous information to capture the embedding of drugs and targets, and provide assistance for drug development. For the convenience of related researchers, a user-friendly web-server has been established at http://bioinfo.jcu.edu.cn/hgdti.

## Background

Drug-like compounds achieve curative effects through biochemical reactions with in-vivo protein molecules such as enzymes, ion channels, G protein-coupled receptors(GPCR). Due to the incompletely understanding of drug molecules and the diversity of targets, clinical trials for new drug–target interactions (DTIs) have become time-consuming and required costly investments. Identifying new DTIs through computational approaches can significantly reduce the time and cost required for drug discovery or relocation compared with biochemical experimental methods [1].

At present, the calculation methods for identifying DTIs can be divided into three categories, ligand-based, docking simulation, and chemogenomic approaches. Ligand-based methods [2], like Quantitative Structure-Activity Relationship (QSAR), predict the interaction by comparing the similarity of new ligands and known proteins ligands. However, ligand-based methods often perform poorly when the number of known binding ligands for proteins is insufficient. Docking simulation methods [3] require the simulation of the three-dimensional structure of proteins. Such methods are inapplicable when numerous proteins with unknown 3D structure. Chemical genomics methods [4] attempt to take advantage of the interaction, similarity and association between drugs, proteins and other biomarkers (e.g. disease and side-effect) to construct a unified chemical genome space [5]. Moreover, these approaches build predictors based on machine learning to discover unknown interactions between drugs and proteins. These predictors are based on the “guilt by association” assumption where similar drugs may share similar targets and vice versa.

Previously, various models utilized machine learning methods to identify DTIs [6], such as nearest neighbor methods [7, 8], matrix factorization methods [9], semi-supervised learning methods [10]. These methods all directly use the molecular structure information of drugs and the sequence information of targets as input features to construct an algorithm model to classify DTIs. Mei et al. [11] advanced the bipartite local model (BLM) by adding a neighbor-based interaction-profile inferring (NII) procedure (called BLMNII), which learnt interaction features from neighbors to preprocess training data. NetLapRLS [12] applied Laplacian regularized least-square (RLS) and integrated information kernels from chemical space, genomic space and drug–protein interaction into the prediction framework. MSCMF [13] incorporated multiple similarity matrices, including the similarity of chemical structure, genomic sequence, ATC, GO and PPI network, to regulate the DTI network. Recently, deep learning technology has been widely used, and many methods have achieved substantial performance improvements in DTIs by constructing complex neural networks [13–15]. DeepDTA [16] employed CNN blocks to learn representations from the raw protein sequences and SMILES strings and combine these representations to feed into a fully connected layer block. Lee et al. [17] constructed a novel DTI prediction model to extract local residue patterns of target protein sequences using a CNN-based deep learning approach.

Due to the development of feature extraction technology, many excellent models with higher predictive capacity have emerged to cope with the identification problem of drug compound and protein sequence [18–21]. In addition to drug molecular structure and protein sequence data, drug side effects [22], drug-disease association and target-disease association [23] can also be used to improve DTI networks and discover the relationship between drugs and proteins from diverse perspectives. In DTINet [24] and NeoDTI [25], integrating heterogeneous features from heterogeneous data sources can improve the DTI predictive ability of model. However, there are still some unsolved problems concerning these method. In DTINet, separating feature processing and model training may lose the optimal solution. NeoDTI utilized random vectors to initialize heterogeneous node features may reduce prediction precision. Besides, it adversely affects the prediction result when NeoDTI fuses neighbor features and ignores the importance of each neighbor. Recently, the theory of graph neural network (GNN) [26] has matured, and the algorithm framework has gradually enriched, including GCN (Graph Convolution Networks), GAT (Graph Attention Networks) [27], GAE (Graph Autoencoders) [28]. Zhang et al. [29] proposed a heterogeneous graph neural network (HetGNN), which applies a series of aggregation operations to heterogeneous neighbors to obtain the ultimate node embedding. This inspired us to build our own model for discovering new DTIs.

In this paper, we present HGDTI model, a heterogeneous graph neural network for predicting DTI. Firstly, in the pre-processing step, we sample negative pairs from unknown DTI pairs by employing negative sampling technology. Then, HGDTI uses LSTM to abstract content of the node (e.g. drug, protein, disease, and side-effect), and extracts the final embedding of drugs and proteins by aggregating the contents of heterogeneous neighbors. Finally, the obtained drug and protein embeddings are used to predict DTI through a fully connected neural network. The entire learning and prediction process is an end-to-end workflow. Hence, it is possible to obtain the feature representation of drugs and targets closest to the DTI network. Through comprehensive tests, we compare the performance of DTI prediction between HGDTI and other state-of-the-art predictors. In addition, the robustness and extendability of HGDTI are inspected by testing partial heterogeneous networks. Overall, HGDTI can integrate more heterogeneous data sources to provide more accurate results for DTI prediction, which may also provide a better solution for drug discovery and repositioning.

## Methods

### DTI problem formulation

**Fig. 1:**
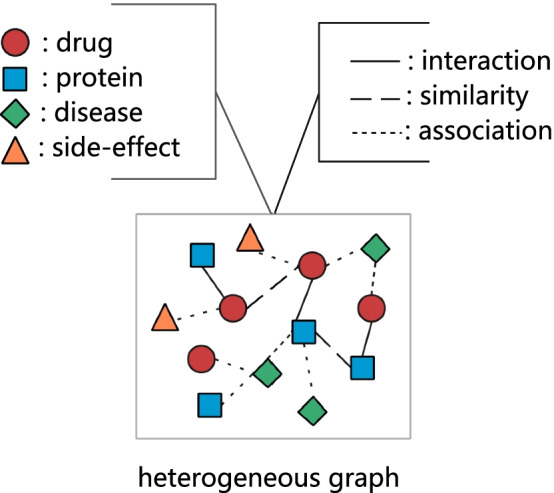
Dataset: a heterogeneous graph with different nodes and edges

In this work, the dataset is a heterogeneous graph composed of various nodes and edges. Nodes include drugs, proteins, diseases, and side effects. Edges include interactions, similarities, and associations. Our model learns embedded representations of drugs and proteins from this graph to predict DTIs. Next, the definition of heterogeneous graph is given.

Definition HG (Heterogeneous Graph). HG is defined as an undirected graph $G = \left( V, E, O_V, R_E\right)$, where *V* is the node set, *E* is the edge set, the object type of each node $v \in V$ belongs to the object type set $O_V$, the relation type of each edge $e \in E$ belongs to the relation type set $R_E$. Besides, we define that $C\left( v\right) \in {\mathbb {R}}^{\left| V\right| \times dim}$ (*dim*: feature dimension) maps the initial feature set of nodes, $F\left( v\right) \in {\mathbb {R}}^{\left| V\right| \times dim}$ indicates final embeddings.

The node type set $O_V$ includes drug, target, side-effect and disease. The link type set $R_E$ is composed of drug-similarity-drug, drug-interaction-drug, protein-similarity-protein, drug-interaction-protein, drug-association-disease, etc., total 8 types (as shown in Fig. 1, also available See “Datasets” section). It is noted that all nodes are connected via interaction, similarity, or association edges with non-negative weight $W_e$. Among that, interaction edge or association edge with value 1. In addition, the edge weight between two “unrelated” nodes is set to 0, such as unknown DTIs. Besides, there are two edges connected between two nodes simultaneously. For example, two drugs are connected through the drug-similarity-drug edge and drug-interaction-drug edge.

### Embedding learning

In the graph network, the embedding learning model is to use the topology structure and the content information of the node in the network to obtain the final representation of the node. For example, DeepWalK [30], node2vec [31] and metapath2vec [32] employ random walk strategies to get the context sequence of the node in the network and learn node embedding with the help of word2vec [33]. struc2vec [34] leveraging local network structure information to differentiate node representation. GCN [26], the graph neural network version of CNNs, aggregates local (i.e. adjacency) context information of the node through a series of convolution operations. Different from the random walk strategy and simple convolution operation in the above methods, HGDTI only considers the first-order relationship (i.e. direct relationship) between nodes and convolves the information of adjacent neighbors. Moreover, in order to distinguish the importance of different types of neighbors, different weights are set for different types of neighbors during the aggregation process.

### Pre-processing

In the actual training scenario, the number of known DTIs is much lower than unknowns. Such an extremely unbalanced dataset brings incredible difficulty to DTI network prediction. A solution is to employ random sampling to construct negative samples from unknown DTIs. Nevertheless, this way may reduce the accuracy of prediction and treat unknown drug–target pairs that exist possible interactions as non-interactions. A previous research by Liu et.al. [35] demonstrated the correctness of negative samples sampling method directly affected the prediction performance. Recently, Eslami et.al. [15] also utilized a similar method to preprocess the negative sample dataset and obtained remarkable experimental results. Similarly, we screen out reliable negative samples. The screening basis is that drugs that are not similar to or do not interact with all drugs corresponding to the target in known DTIs are unlikely to interact with the target and vice versa. Firstly, we denote the drug set *D* and the target set *T*, sort out the target list $T_{d_i}\left( d_i \in D\right)$ corresponding to each drug $d_i$ and the drug list $D_{t_j}\left( t_j \in T\right)$ corresponding to each protein $t_j$ from known DTIs, respectively. Secondly, give drug matrix $A \in {\mathbb {R}}^{\left| D\right| \times \left| D\right| }$ representing DDS matrix (i.e. drug-drug chemistry similarity matrix), and target matrix $B \in {\mathbb {R}}^{\left| T\right| \times \left| T\right| }$ representing PPS matrix (i.e. protein–protein sequence similarity matrix). Then, define reliable score $s_{ij}$ of the drug–target pair $\left( d_i-t_j\right)$ in unknown DTIs. Define $s^{DT}_{ij} = \sum _{t_k\in T_{d_i}} B_{t_jt_k}$, that sum up the similarity between the target list $T_{d_i}$ that interact with drug $d_i$ and target $t_j$. Similarly, define $s^{TD}_{ji} = \sum _{d_k\in D_{t_j}} A_{d_id_k}$, which sums up the similarity between the drug list $D_{t_j}$ that interact with target $t_j$ and drug $d_i$. Finally, a reliable score $s_{ij}$ between drug $d_i$ and protein $t_j$ is computed as:

1 $$\begin{aligned} s_{ij} = e^{-\left( s^{DT}_{ij}+s^{TD}_{ji}\right) } \end{aligned}$$

The negative candidate pairs are arranged in descending order according to the reliable score calculated by the above formula, and the high score is selected as the reliable negative DTIs. Sample a certain number of unknown DTIs as negatives and known DTIs as positives to form the complete data set for subsequent model training and testing.

### Representing drug molecules with the 2D molecular fingerprint

HGDTI leverages the molecular fingerprint approach to extract the initial feature of the drug, which is frequently employed in drug-related prediction problems [36–39]. Molecular fingerprint is a method of binary coding of molecular structure to describe the presence or absence of particular substructures. Xiao et.al. [37] has given a crystal clear description of how to obtain the molecular fingerprint of the drug compound, and hence there is no need to repeat here. It is noted that we download the SMILES file of the drug from https://go.drugbank.com/. Drug molecular fingerprint $C_{{\widetilde{d}}}$ is represented as a 256-digit hexadecimal string. In particular, the optimal dimension *dim* of drug feature $C_d$ in HGDTI is 128 (See “Hyperparameter Selection” section). Therefore, the dimension of $C_d$ needs to be reduced. Generally, the feature size reduction methods include embedding and fully connection. Here the average approach is adopted. Formally, the content feature of drug *d* is computed as follows:

2 $$\begin{aligned} C_d = \frac{C_{{\widetilde{d}}}[0:127] + C_{{\widetilde{d}}}[128:255]}{2} \end{aligned}$$

where $C_{{\widetilde{d}}}[0:127]$ and $C_{{\widetilde{d}}}[128:255]$ stand for the pre-128 bits and the post-128 bits of $C_{{\widetilde{d}}}$ respectively.

### Representing protein sequences with pseudo amino acid composition

Pseudo amino acid composition(PseAAC) [40] can capture the amino acid composition information of protein sequence and preserve the sequence-order information. Above all, there are ten kinds of physical and chemical properties representing protein properties [37] to convert protein sequences into real strings. HGDTI chooses hydrophobicity, hydrophilicity and side-chain mass as three types of amino acid properties, and the dimension of protein feature vector $C_{{\widetilde{t}}}$ is set to 64. For the specific calculation method, refer to PseAAC or visit https://ifeature.erc.monash.edu/. Finally, we elevate the optimal dimension of protein feature $C_t$ to 128, and the duplicate concatenation method is adopted. Thus the content feature of target *t* is formulated as:

3 $$\begin{aligned} C_t = C_{{\widetilde{t}}} \oplus C_{{\widetilde{t}}} \end{aligned}$$

The operator $\bigoplus$ denotes concatenation.

### The workflow of HGDTI

**Fig. 2:**
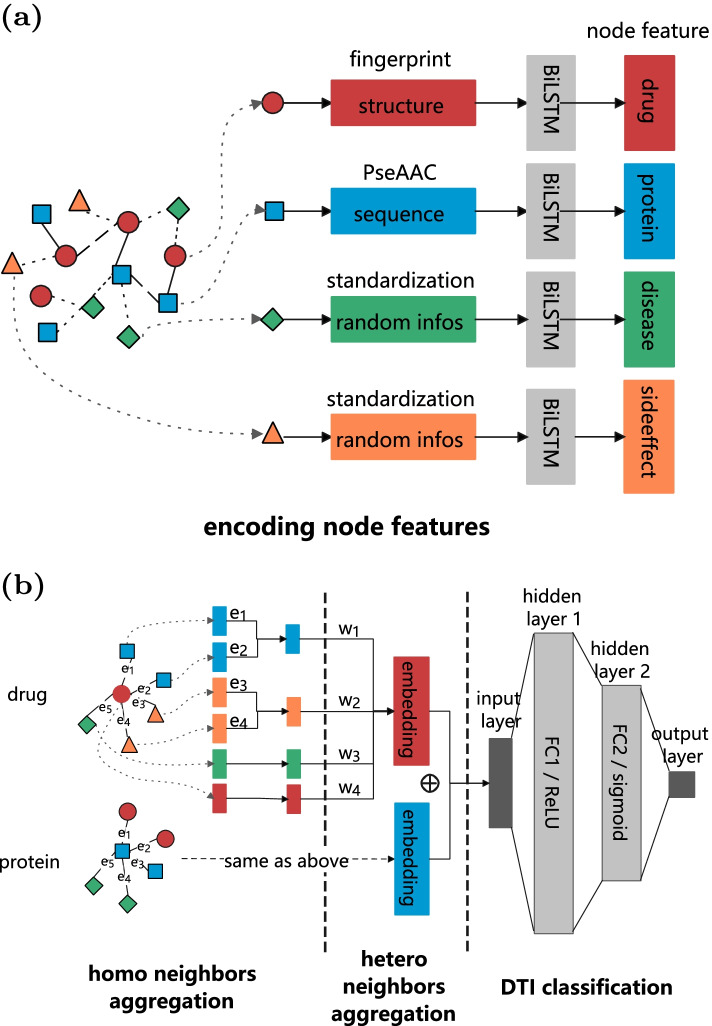
**a** Encode each node feature via BiLSTM. **b** Aggregate neighbors to obtain drug and protein embedding, predict label via a two-layers neural network, finally optimizes the model via a cross-entropy loss

HGDTI consists of the following four main steps: (1) node features encoding; (2) homogeneous neighbors aggregation; (3) heterogeneous neighbors aggregation; (4) predictor training process. Steps(1-3) are to learn the node embeddings that encode both heterogeneous neighbors and itself characteristic contents. Step(4) is a deep neural network classifier, which is used to predict DTIs by training the node embedding to obtain a 0-1 threshold. Next, we will introduce the algorithm formula for each step in detail. The whole process is illustrated in Fig. 2.

Step 1: Node Features Encoding. We have defined the initial features of nodes as $C\left( v\right)$, where the drug feature $C_d$ is extracted from the molecular fingerprint, the protein feature $C_t$ is extracted from PseAAC, the disease and side-effect features are represented by parameterized 0-1 standardized stochastic vector [25] to learn the optimal representation and speed up convergence. In this step, we define a submodule based on bi-directional LSTM (Bi-LSTM) [41] to capture “deep” feature interactions and obtain more abstract nonlinear expressions. The feature encoding for node *v* is defined as:

4 $$\begin{aligned} f_1\left( v\right) = \overrightarrow{LSTM}\left\{ C\left( v\right) \right\} \oplus \overleftarrow{LSTM}\left\{ C\left( v\right) \right\} \end{aligned}$$

where $f_1(v) \in {\mathbb {R}}^{dim \times 1}$ (*dim*: feature dimension), the operator $\oplus$ denotes concatenation. Bi-LSTM block treats each one-dimensional input (vector) as a sentence with only one word ($1 \times dim$ tensor). Overall, the above formula uses Bi-LSTM to extract the general content embedding of *v*, as illustrated in Fig. 2a. Note that single feature $C\left( v\right)$ can flexibly extend the model by adding other features (e.g. the physical and chemical properties of drugs [42], the PSSM profile of proteins [43]) for weighted average. In particular, four Bi-LSTM models are utilized to extract the content of different types of nodes respectively.

Step 2: Homogeneous Neighbors Aggregation. In this step, we design a submodule that aggregates heterogeneous adjacent node features. $N_r\left( v\right) = \left\{ u, u\in V, u\ne v, r\in R_E \right\}$ denotes neighbor set that links to node *v* via edges of type *r*. Then, we employ an aggregated function $G^r$ to fuse features of $u \in N_r\left( v\right)$. $G^r$ is a weighted summation that is not alike from neighbors aggregation approach of HetGNN [29], which treats all edges as equal. Formally, the aggregated embedding of $N_r\left( v\right)$ is defined as:

5 $$\begin{aligned} G^r\left( v\right) = \sum _{u \in N_r\left( v\right) , e = \left( v, u, r\right) , r \in R_E} W_e \frac{f_1\left( u\right) }{M^r\left( v\right) } \end{aligned}$$

where $G^r \in {\mathbb {R}}^{dim\times 1}$ (*dim*: feature dimension), $f_1\left( u\right)$ is feature encoding of node *u* which is calculated by step(1), $W_e$ is a non-negative weight which represents a score of edge *e*. $M^r \left( v\right) = \sum _{u \in N_r\left( v\right) , e = \left( v, u, r\right) } W_e$ stands for a normalization term. To be more specific, *r*-type aggregated embedding for node *v* is summed by same type neighbors feature to multiply by ratio which is the normalized weight (e.g. $\frac{W_e}{M^r\left( v\right) }$) with respect to edges of type *r*.

Step 3: Heterogeneous Neighbors Aggregation. Continue to the previous step, we have got the aggregated embedding $G^r\left( v\right)$ with respect to edge-type *r* for node *v*. Taking into account that heterogeneous nodes have different degrees of impact on the final embeddings, we employ the attention mechanism [27] to incorporate the aggregated embedding $G^r\left( v\right)$ with the initial feature $C\left( v\right)$ of node *v*. Formally, the final embedding of node *v* is formulated as follow:

6 $$\begin{aligned} F\left( v\right) = \alpha ^v C\left( v\right) + \sum _{r \in R_E} \alpha ^r G^r\left( v\right) \end{aligned}$$

where $F\left( v\right) \in {\mathbb {R}}^{\left| V\right| \times dim}$ ($\left| V\right|$: node size, *dim*: feature dimension), $\alpha ^*$ (e.g. $\alpha ^v$, $\alpha ^r$) indicates influence level for the final embeddings. Then, we define $\varphi \left( v\right)$ that stands for $C\left( v\right)$ and $G^r\left( v\right)$, the *i*-th weight factor $\alpha ^i = \frac{exp\left\{ LeakyReLU\left( u^T \varphi _i\right) \right\} }{\sum _{\varphi _j \in \varphi \left( v\right) } exp\left\{ LeakyReLU\left( u^T \varphi _j\right) \right\} }$, $\alpha ^i \in \alpha ^*$. Among them, *LeakyReLU* denotes a leaky version of a Rectified Linear Unit, $u \in {\mathbb {R}}^{2dim\times 1}$ is the attention parameter.

Our task is to predict the drug–target interaction. In the final prediction step, only the final embeddings of drug and target are involved. Therefore, node *v* in steps(2-3) refers to drugs and targets.

Step 4: DTI Classification. To determine whether there is an interaction between the drug–target pair, we employ a fully connected neural network to train the drug embedding $F_d\left( u\right)$ and the protein embedding $F_t\left( v\right)$ and predict DTIs. Thus, the predict probability function *O* is defined as follow:

7 $$\begin{aligned} O = sigmoid\left( FC_2\left( ReLU\left( FC_1\left( F_d\left( u\right) \oplus F_t\left( v\right) \right) \right) \right) \right) \end{aligned}$$

where $FC_1$ and $FC_2$ form a two-layer fully connected neural network that performs a linear transformation on embeddings, *ReLU* (Rectified Linear Unit) indicates nonlinearity capability of the model. The operator $\oplus$ denotes concatenation between the drug embedding and the protein embedding to obtain $2\times dim$ dimension embedding, which is the input of first layer $FC_1$. Specifically, $FC_1$ has *dim*/2 neurons which are connected to each dimension of the input embedding, $FC_2$ that the final output layer contains only one neuron corresponding to output result which is fully connected to the previous layer, *sigmoid* stands for a nonlinear activation function that projects from the result of a final layer onto DTI probability. Steps(2-4) are shown in Fig. 2b.

At last, we adopt cross-entropy loss function that calculates the difference between DTI probability and drug–target pair label.

In general, all the above steps can be trained through an end-to-end manner by performing *Adam* optimizer [44] and 0.001 learning rate to minimize the final loss function and update the model parameters. We repeat the training iterations until the change between two consecutive iterations is less than the threshold. The entire framework is implemented on the PyTorch platform and GPU hardware.

## Data and experiment

### Datasets

**Table 1:** Dataset statistic of each comparison experiment

	Positive	Training set	Validation set	Test set
test a	1923	18085	952	2116
test b	968	9103	480	1065
test c	1557	14643	771	1713
test d	1872	17605	927	2060
test e	1126	10589	558	1239
test f	1551 + 372 (test)	16207	854	4092

The datasets are collected from previous research [24], include 4 types of nodes and 8 types of edges. Specifically, 708 drugs, 1,923 known DTIs as well as drug–drug interaction network have been extracted from DrugBank (Version 3.0) [45]. 1,512 proteins and protein–protein interaction network have been extracted from the HPRD database (Release 9) [46]. 5,603 diseases, drug-disease association and protein-disease association networks have been extracted from the Comparative Toxicogenomics Database [47]. 4,192 side-effects and drug-side-effect association network have been extracted from the SIDER database [48]. In addition, 364 side-effects and 161 diseases are isolated. Besides, we adopt two similarity information, a drug-structure similarity network (i.e. a pair-wise chemical structure similarity network measured by the dice similarities of the Morgan fingerprints with radius 2, which have been computed by RDKit [49]), and a protein sequence similarity network (which have been obtained based on the pair-wise Smith-Waterman score [50]). The datasets have been utilized in previous researches [15, 25]. As shown in the statistics in Table 1, tests a-f same as in NeoDTI [25] corresponds to Figs. 4 and 5.

### Reliable negatives

**Fig. 3:**
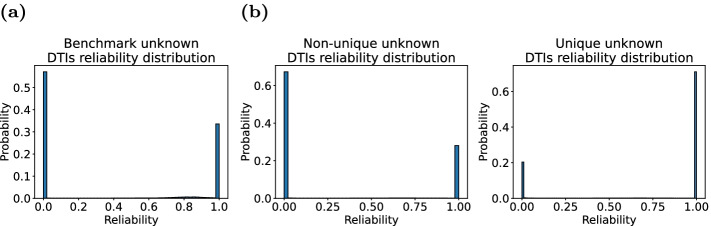
Unknown DTIs reliability distribution. **a** Benchmark unknown DTIs reliability distribution. **b** Non-unique and unique unknown DTIs reliability distribution

In the original dataset, the vast majority of DTIs are unknown, including potential DTIs and non-DTIs. Unlike the previous model which treats all unknown DTI pairs as negative samples, we consider selecting the “correct” unknown DTI pairs as negative samples as much as possible. We employ negative sampling technique (See “Pre-processing” section) to calculate reliable scores between drugs and targets, and divide reliable negative samples according to the distribution of reliable scores of drug–target pairs (Fig. 3). As the figure, the reliable scores of unknown DTIs are mainly concentrated around 0 score and 1 score. Combined with specific numerical analysis, we choose DTI with a reliable value greater than 0.1 as a negative sample, which is equivalent to nearly half of the unknown in benchmark (Fig. 3a), 30% in non-unique and 80% in unique (Fig. 3b).

**Fig. 4:**
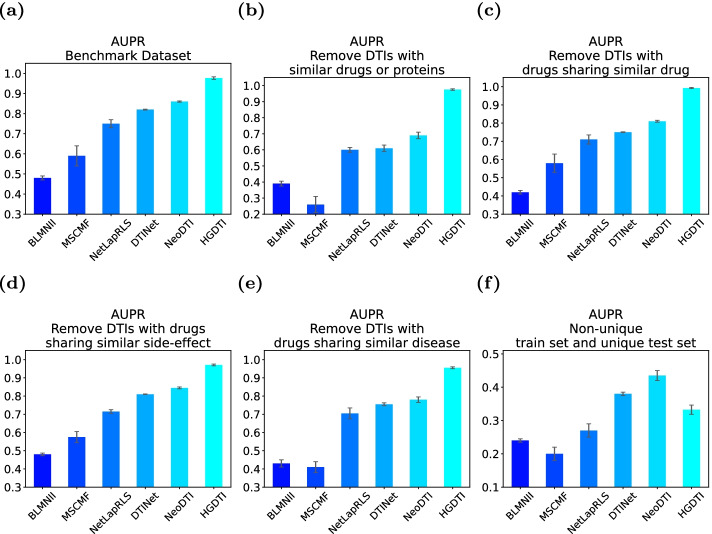
Comparison results of HGDTI with other state-of-the-art models in several exploratory experiments in terms of the AUPR scores. **a** A 10-fold cross-validation test in which the ratio between positive and negative samples is set to 1 : 10. **b**–**e** Ten-fold cross-validation with positive: negative ratios $=1 : 10$ on several scenarios of removing redundancy in data. **b** Remove DTIs with similar drugs and proteins. **c** Remove DTIs with drugs sharing similar drug interactions. **d** Remove DTIs with drugs sharing similar side-effects. **e** Remove DTIs with drugs sharing similar disease. **f** Non-unique train set and unique test set. All results are summarized over 10 trials and expressed as mean ± standard deviation

**Fig. 5:**
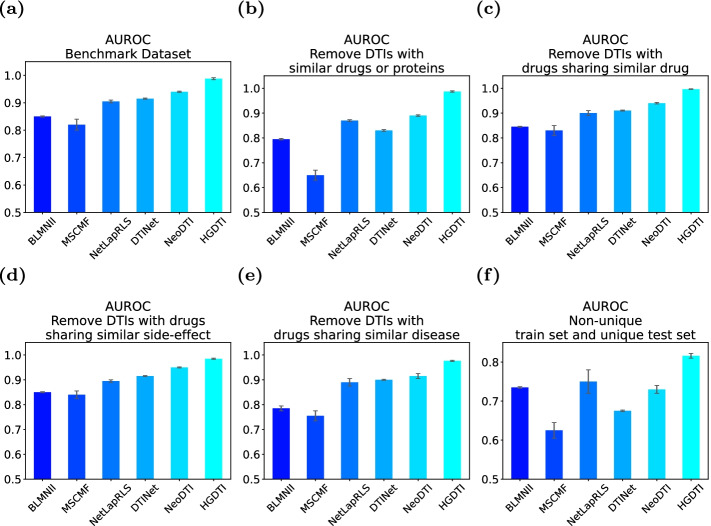
Comparison results of HGDTI with other state-of-the-art models in several exploratory experiments in terms of the AUROC scores. **a** A 10-fold cross-validation test in which the ratio between positive and negative samples was set to 1 : 10. **b**–**e** Ten-fold cross-validation with positive: negative ratios $=1 : 10$ on several scenarios of removing redundancy in data. **b** Remove DTIs with similar drugs and proteins. **c** Remove DTIs with drugs sharing similar drug interactions. **d** Remove DTIs with drugs sharing similar side-effects. **e** Remove DTIs with drugs sharing similar disease. **f** Non-unique train set and unique test set. All results were summarized over 10 trials and expressed as mean ± standard deviation

### HGDTI yields significant capability for DTIs prediction

For the sake of comparing HGDTI with the previous state-of-the-art DTI prediction methods, we use the same dataset and the 10-fold cross-validation method. To mimic this scenario that only a minimal number of drug–target pairs are known DTIs in the practical situation, we sample all positive samples (known DTIs) and negative samples, which are selected based on the method explained in “Reliable negatives” section, in which negative samples are 10 times that of positive samples. During the experiment, the dataset will be cross-cut by hierarchical sampling to ensure that the proportions of various samples in the training set and test set are the same as the original dataset. The dataset is divided into 10 non-overlapping subsets according to the ratio (i.e. 1:10) of positive and negative samples in the original data set, 9 subsets are used as the training set and the remaining 1 subset is used as the test set. Like other predictive methods, we employed the Area Under Receiver Operating Characteristic (AUROC) curve and Area Under Precision-Recall (AUPR) curve to evaluate prediction performance for all methods. In general, ROC curves present the trend between true positive rate (TPR) and false positive rate (FPR), and PR curves reveal the trend between precision and recall using several classification thresholds. AUPR is more sensitive than AUROC for extremely skewed datasets. Therefore, the predictive ability of model can be better explained in such a scenario. Since random sampling will cause jitter in the prediction results, we randomly select 10 sets of samples through 10 fixed second-level random seeds generated from a first-level random seed “10”. The second-level random seeds are shown in Table 2. The final result is summarized over 10 trials and expressed as mean ± standard deviation.

**Table 2:** Second-level random seed list

	No.1	No.2	No.3	No.4	No.5
Seed	265	125	996	527	320

	No.6	No.7	No.8	No.9	No.10
Seed	369	123	156	985	733

We compare the performance of HGDTI with six predictive models, including NeoDTI [25], DTINet [24], MSCMF [13], NetLapRLS [12] and BLMNII [11]. The result of the comparison shows that HGDTI remarkably outperforms other models, with 11.1% higher AUPR and 4.5% higher AUROC than the second-best method (Figs. 4a, 5a). DTINet generates low-dimensional features representing the structure of nodes in context through a network diffusion algorithm (random walk with restart, RWR). HGDTI adopts the fingerprint features of drug molecules and the PseAAC features of proteins, and enhances feature learning through the neighborhood aggregation of nodes. Comparing with NeoDTI, HGDTI uses weighted aggregation of heterogeneous neighbors and utilizes reliable negative samples. The process of searching the hyperparameter of feature dimension in these baseline methods can be found in “Hyperparameter selection” section.

The original dataset may contain approximate samples (i.e. sharing homologous proteins and similar drugs between know DTIs), which may affect the veracity of the predictive power by easy predictions. To explore this issue, we perform the following additional tests (Figs. 4b–e, 5b–e): (1) the removal of DTIs with similar drugs (i.e. drug chemical structure similarities > 0.6) or homologous proteins (i.e. protein sequence similarities > 0.4); (2) the removal of DTIs with drugs sharing similar drug interactions (i.e. Jaccard similarities > 0.6); (3) the removal of DTIs with drugs sharing similar side-effects (i.e. Jaccard similarities > 0.6); (4) the removal of DTIs with drugs or proteins sharing similar diseases (i.e. Jaccard similarities > 0.6). In the above experimental scenarios, we adopt the same positive and negative sample ratio and the uniform 10-fold cross-validation method. All test results demonstrate that HGDTI still remarkably outperforms other prediction methods after the removal of redundant samples, which also certifies the stability of HGDTI.

In addition, we also conducted comparative experiments on “unique” data, in which drugs interact with only one target and vice versa. In that, the unique DTIs prediction lacks sufficient neighbors. To assess the performance of DTIs prediction methods in this scenario, we split the dataset into non-unique DTIs and unique DTIs, which are used in the training phase and the test phase respectively, and the ratios between positive and negative remain unchanged. We detect that HGDTI is unsatisfactory in terms of AUPR (Fig. 4f), which indicates that HGDTI is not suitable for improving model performance by capturing rich neighborhood information in sparse networks.

It can be seen that discrete nodes that are more extreme than “unique” have worse prediction results, which is also the limitation of graph neural networks. Therefore, for new drugs and new targets that are not in the graph HG, HGDTI cannot aggregate the multi-source information around the node, resulting in unsatisfactory predictive performance.

**Fig. 6:**
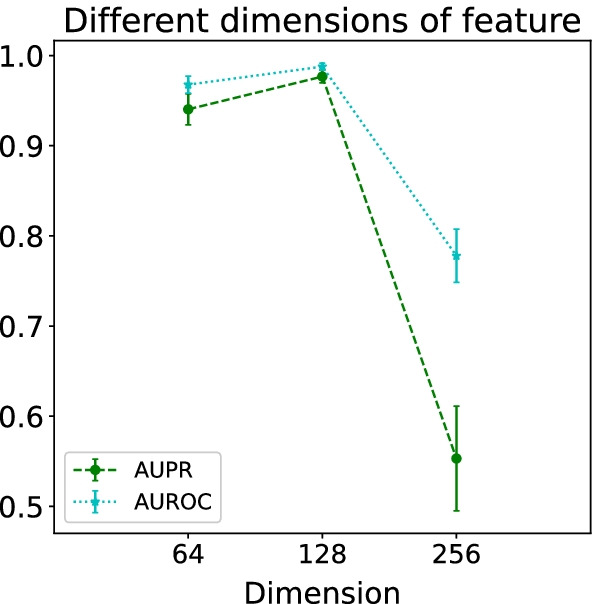
Optimal dimension of feature. All results were summarized over 10 trials and expressed as mean ± standard deviation

**Fig. 7:**
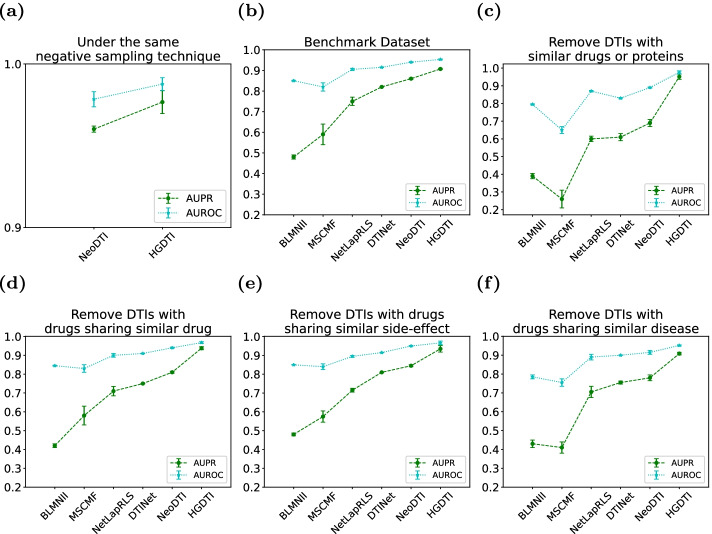
Verify the impact of negative sampling technique (NS) in terms of the AUPR and AUROC scores. A 10-fold cross-validation test in which the ratio between positive and negative samples is set to 1 : 10. **a** The second-best NeoDTI with NS. **b**–**f** HGDTI without NS in several exploratory experiments. All results are summarized over 10 trials and expressed as mean ± standard deviation

**Fig. 8:**
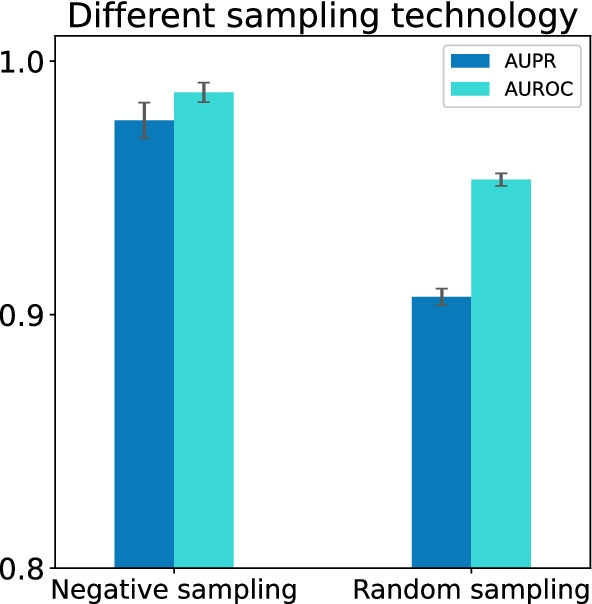
Comparison result of negative sampling with random sampling in terms of the AUPR and AUROC scores. All results were summarized over 10 trials and expressed as mean ± standard deviation

### Hyperparameter selection

All node features adopt a uniform dimension $d \in {64, 128, 256}$. To determine the optimal representation dimension of feature, we randomly divide the training set into 5% as the validation set to select the best hyperparameter. The result is shown in Fig. 6. When *d* = 64, 128 and 256, the corresponding AUPR scores were 0.899, 0.961 and 0.585 respectively, while the corresponding AUROC scores were 0.946, 0.979 and 0.795 respectively. Consequently, HGDTI has the best prediction effect and the smallest variance result when d = 128.

### The rationality of negative sampling technique

In order to prove that the superiority of the HGDTI algorithm is not contributed by the negative sampling technique, we compare the second-best NeoDTI with HGDTI under the condition of the negative sampling technique. As presented in the results, HGDTI outperforms NeoDTI by 1.7% in terms of AUPR and 0.9% in terms of AUROC (Fig. 7a). At the same time, we test the performance of HGDTI without negative sampling technology on several scenarios (Fig. 7b–f). In the first test, we observe a significant improvement (4.5% in terms of AUPR and 1.3% in terms of AUROC) over the second-best NeoDTI. These results indicate that under the same sampling conditions, the power of HGDTI to identify DTI is better than other models, and negative sampling technology can further narrow the prediction range of model.

To study the impact of negative sampling technology on the classification ability of HGDTI, we further achieve model’s DTIs prediction results using random sampling. As expected, model’s ability to identify DTIs dropped prominently by 6.9% in terms of AUPR and 3.4% in terms of AUROC (Fig. 8). The importance of negative sampling technology is self-evident.

**Fig. 9:**
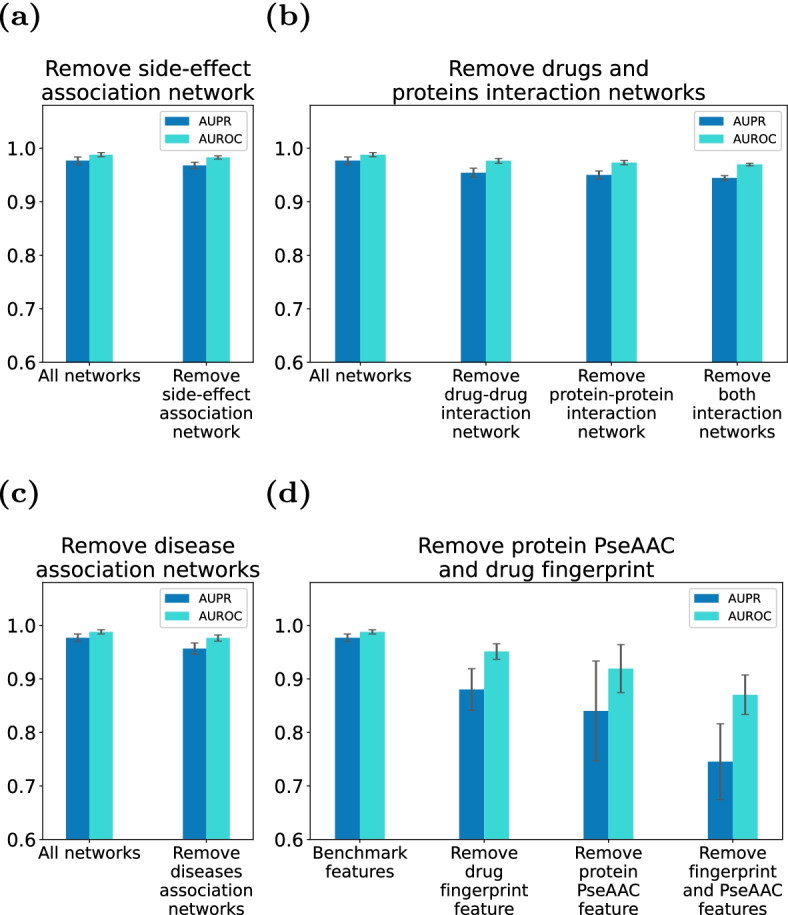
Remove HGDTI’s drug or target-related information can reduce predictive performance. **a** Remove drug-side-effect association network. **b** Remove drugs and proteins interaction networks. **c** Remove disease association networks. **d** Remove drug fingerprint and protein PseAAC. All results are summarized in 10 trials and expressed as mean ± standard deviation

**Fig. 10:**
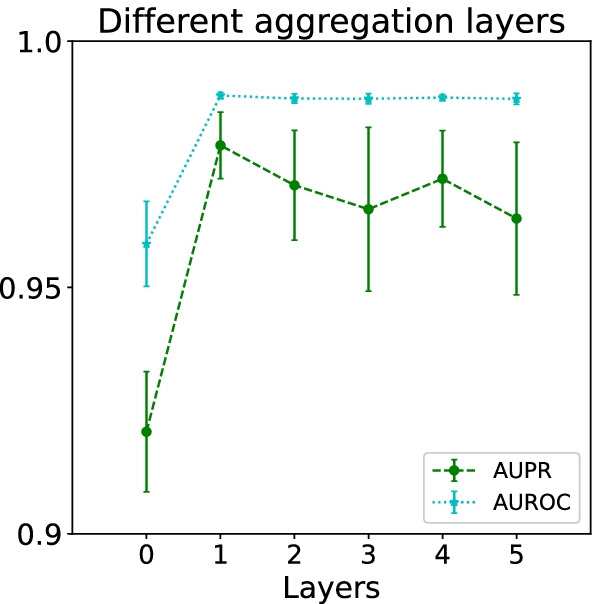
Comparison result of different aggregation layers in terms of the AUPR and AUROC scores. All results were summarized over 10 trials and expressed as mean ± standard deviation

### Robustness of HGDTI

In the following section, we would like to discuss the robustness of model and the correctness of design. Above all, we further explore the influence of integrating multiple heterogeneous data on DTIs prediction. The experimental data is formed by deleting heterogeneous networks on the basis of the benchmark dataset, and the experimental evaluation method remains unchanged. We first remove the side-effect network, and model prediction results decrease slightly with 0.9% in terms of AUPR and 0.5% in terms of AUROC (Fig. 9a). Then contrast the experimental results of removing the drug or protein interaction network in the heterogeneous network (Fig. 9b). Subsequently, the disease network is removed from the benchmark dataset, and the evaluation metric is significantly reduced by 2.0% in terms of AUPR and 1.2% in terms of AUROC (Fig. 9c). The contrast of these experiments indicates that the fusion of different individual networks can more accurately express the characteristics of drugs and targets and improve the performance of DTIs prediction.

In the benchmark dataset, we find that the effective representation of the node itself is missing. In order to complement the features of drugs and proteins, HGDTI introduces drug molecular fingerprint features (“Representing drug molecules with the 2D molecular fingerprint” section) and protein pseudo-amino acid composition information (“Representing protein sequences with pseudo amino acid composition” section). We further investigate the effect of these features on the model. The experimental results show that the absence of molecular fingerprint information leads to 9.7% reduction in the AUPR metric and 3.7% decrease in the AUROC metric, and the absence of pseudo-amino acid component results in loss with 13.7% in the AUPR metric and 6.9% in the AUROC metric (Fig. 9d), which sufficiently proves the contribution of molecular fingerprint and pseudo-amino acid component to the predictive ability of HGDTI.

According to Henaff’s conclusion [51] that higher layers have lower performance, we only construct one layer of neighborhood aggregation. To illustrate the correctness of the structural design, we experiment with the effect of various neighborhood extents on predictive capability. The comparison (Fig. 10) reveals that the aggregation operation significantly improves the performance, but the results decrease slightly as the aggregation layer deepens. The fifth-order aggregation has only more than 1% AUPR difference.

## Conclusion

We have proposed a DTI prediction methodology, called HGDTI, to learn the embedding of drugs and targets hidden in various heterogeneous network and input into a fully connected neural network to predict DTIs. The entire framework is divided into a feature learning neural network and a label prediction neural network. By optimizing the parameters of HGDTI through an end-to-end approach, the former can capture more reliable features, and the latter can predict closer labels. After several realistic test scenarios, it is proved that HGDTI is superior to other methods in terms of prediction performance and can integrate more heterogeneous networks to improve prediction accuracy. Moreover, negative sampling technology can further narrow the prediction range. In general, HGDTI can be utilized as an excellent tool for computational drug discovery and drug repositioning.

## Data Availability

The dataset, code and materials used in this project can be found in: http://bioinfo.jcu.edu.cn/hgdti, https://drive.google.com/drive/folders/1go6xZXRR6gFosogrGzNkzWiEzD4WSy9Z?usp=sharing

## Abbreviations

DTI: Drug–target interaction
Bi-LSTM: Bi-directional-long short-term memory
QSAR: Quantitative structure-activity relationship
BLM: bipartite local model
RWR: Random walk with restart
ReLU: Rectified linear unit
AUROC: Area under receiver operating characteristic
AUPR: Area under precision-recall
TPR: True positive rate
FPR: False positive rate
